# Supplementary material for: Knapping tools in Magdalenian contexts: New evidence from Gough’s Cave (Somerset, UK)
Source: PLoS One. 2021 Dec 23;16(12):e0261031. doi: 10.1371/journal.pone.0261031 (PMC8699705; doi:10.1371/journal.pone.0261031)
Supplement: Attachment — Submitted filename: Response to Reviewers.docx [file pone.0261031.s001.docx]

**Journal Requirements**:

Please review your reference list to ensure that it is complete and correct. If you have cited papers that have been retracted, please include the rationale for doing so in the manuscript text, or remove these references and replace them with relevant current references. Any changes to the reference list should be me­ntioned in the rebuttal letter that accompanies your revised manuscript. If you need to cite a retracted article, indicate the article’s retracted status in the References list and also include a citation and full reference for the retraction notice.

When submitting your revision, we need you to address these additional requirements.

1. Please ensure that your manuscript meets PLOS ONE's style requirements, including those for file naming. The PLOS ONE style templates can be found at 
<https://journals.plos.org/plosone/s/file?id=wjVg/PLOSOne_formatting_sample_main_body.pdf> and 
<https://journals.plos.org/plosone/s/file?id=ba62/PLOSOne_formatting_sample_title_authors_affiliations.pdf>

Authors comments: Style requirements are met in the manuscript.

2. In your manuscript, please provide additional information regarding the specimens used in your study. Ensure that you have reported specimen numbers and complete repository information, including museum name and geographic location.
If permits were required, please ensure that you have provided details for all permits that were obtained, including the full name of the issuing authority, and add the following statement:
'All necessary permits were obtained for the described study, which complied with all relevant regulations.'
If no permits were required, please include the following statement:
'No permits were required for the described study, which complied with all relevant regulations.'
For more information on PLOS ONE's requirements for paleontology and archaeology research, see <https://journals.plos.org/plosone/s/submission-guidelines#loc-paleontology-and-archaeology-research>.

Authors comments: This has been clearly stated in the Section ‘Abbreviations’

3. We note that Figure(s) 1, 12 and 16 in your submission contain [map/satellite] images which may be copyrighted. All PLOS content is published under the Creative Commons Attribution License (CC BY 4.0), which means that the manuscript, images, and Supporting Information files will be freely available online, and any third party is permitted to access, download, copy, distribute, and use these materials in any way, even commercially, with proper attribution. For these reasons, we cannot publish previously copyrighted maps or satellite images created using proprietary data, such as Google software (Google Maps, Street View, and Earth). For more information, see our copyright guidelines: <http://journals.plos.org/plosone/s/licenses-and-copyright>.
 
We require you to either (1) present written permission from the copyright holder to publish these figures specifically under the CC BY 4.0 license, or (2) remove the figures from your submission:
 
1. You may seek permission from the original copyright holder of Figure(s) 1, 12 and 16 to publish the content specifically under the CC BY 4.0 license.  
 
We recommend that you contact the original copyright holder with the Content Permission Form (<http://journals.plos.org/plosone/s/file?id=7c09/content-permission-form.pdf>) and the following text:
“I request permission for the open-access journal PLOS ONE to publish XXX under the Creative Commons Attribution License (CCAL) CC BY 4.0 (<http://creativecommons.org/licenses/by/4.0/>). Please be aware that this license allows unrestricted use and distribution, even commercially, by third parties. Please reply and provide explicit written permission to publish XXX under a CC BY license and complete the attached form.”

Authors comments:

Figure 1: A. Base map drawn by one of the authors by tracing over a standard Europe base map (Source: https://en.wikipedia.org/wiki/Europe#/media/File:Europe_polar_stereographic_Caucasus_Urals_boundary.svg by Ssolbergj; CC BY-SA 3.0). B. Plan re-drawn by the authors based on based on Donovan 1955; Jacobi, 2004, Stringer 2000

Figure 12: Plans re-drawn by the authors based on Donovan 1955; Jacobi, 2004, Stringer 2000.

Figure 16: Figure caption updated with source information: ‘Made with Natural Earth. Free vector and raster map data available at naturalearthdata.com’.

4. We note that Figure(s) 14 and 15 in your submission contain copyrighted images. All PLOS content is published under the Creative Commons Attribution License (CC BY 4.0), which means that the manuscript, images, and Supporting Information files will be freely available online, and any third party is permitted to access, download, copy, distribute, and use these materials in any way, even commercially, with proper attribution. For more information, see our copyright guidelines: <http://journals.plos.org/plosone/s/licenses-and-copyright>.
We require you to either (1) present written permission from the copyright holder to publish these figures specifically under the CC BY 4.0 license, or (2) remove the figures from your submission:

Authors comments: Figures 14 and 15 were both drawn and created by one the authors and have not been previously published.

5. In your Data Availability statement, you have not specified where the minimal data set underlying the results described in your manuscript can be found. PLOS defines a study's minimal data set as the underlying data used to reach the conclusions drawn in the manuscript and any additional data required to replicate the reported study findings in their entirety. All PLOS journals require that the minimal data set be made fully available. For more information about our data policy, please see <http://journals.plos.org/plosone/s/data-availability>.

"Upon re-submitting your revised manuscript, please upload your study’s minimal underlying data set as either Supporting Information files or to a stable, public repository and include the relevant URLs, DOIs, or accession numbers within your revised cover letter. For a list of acceptable repositories, please see <http://journals.plos.org/plosone/s/data-availability#loc-recommended-repositories>. Any potentially identifying patient information must be fully anonymized.

Important: If there are ethical or legal restrictions to sharing your data publicly, please explain these restrictions in detail. Please see our guidelines for more information on what we consider unacceptable restrictions to publicly sharing data: <http://journals.plos.org/plosone/s/data-availability#loc-unacceptable-data-access-restrictions>. Note that it is not acceptable for the authors to be the sole named individuals responsible for ensuring data access.

We will update your Data Availability statement to reflect the information you provide in your cover letter

Authors comments: All data are presented in the main text.

**Reviewer’s** **Comments to the Author**

**Reviewer #1:** In their paper, Bello and collaborators provide a detailed analysis of osseous soft hammers, retouchers and pressure flakers found at Gough’s Cave, a well-studied Magdalenian site that has yielded numerous human remains. This study is particularly relevant both theoretically and methodologically. From a theoretical perspective, ‘expedient’ osseous tools are poorly documented in Upper Palaeolithic context owing to the focus on ‘formal’ tools since the early development of archaeology. Indeed, ‘formal’ tools were easily recognizable and many types played a central role in defining the chrono-cultural framework of the European Upper Palaeolithic. Moreover, ‘expedient’ bone tools are somewhat hard to recognize and many post-depositional processes could result in surface alterations that mimic anthropogenic modifications. Fortunately, studies published in the last three decades have greatly expanded our understanding of these processes and archaeologists are now equipped with a set of tools allowing them to securely make such distinction. From a methodological standpoint, Bello and collaborators provide a rare example of how different microscopic approaches can be combined to support their functional interpretation. Collectively, their results and interpretations provide a new understanding of the complexity inherent to lithic technology during the Magdalenian. I sincerely believe this paper is worthy of publication in PLOS ONE, and recommend only a few minor suggestions, revisions, additions, and clarifications that the authors may want to consider before moving forward. They are listed below.

Lines 89-90: Although it is true that ‘organic (soft) knapping tools appear to be rare in Magdalenian contexts’, this could be also due to the use of Buxus sp. hammers.

Authors comments: This speculation is interesting – unfortunately it cannot be addressed because there are no Palaeolithic wooden hammers. We do, however, mention wood hammers as part of experimental knapping kits. Wood hammers are not included in the list (lines 737-9) of Palaeolithic knapping tools as there are no published examples from a Palaeolithic context.

Lines 161-164: It would be pertinent, in addition to providing the mean value, to include the range of the 14C ages obtain when dating the remains from Gough’s Cave.

Authors comments: We do provide the range of 14C ages and provide a link to the most up-to-date paper with the full set of dates. ‘The dates display a remarkably tightly clustered group with a mean value of 12,600 BP (~14,950 – 14,750 cal BP) indicating that the Magdalenian occupation lasted for as little as two or three human generations [39]’

Lines 166-169: It would be interesting to provide a summary of the evidence for seasonality at Gough’s Cave.

Authors comments: We have added a summary of the evidence for seasonality with the supporting reference (13).

Material and methods: As a general comment, I would appreciate the authors to provide more references on which they based their interpretations on 1) post-depositional alteration, 2) typological attribution, 3) technological identification of use wear traces, 4) breakage patterns.

Authors comments: This has now been added in the text.

Lines 185-214: This section reads more like an overview of “Result and Discussion” rather the “Material and methods”. A more standardized presentation of the Material and methods used in the study would improve the flow of the paper.

Authors comments: We have added a description and references for the methods used to interpret modifications of the faunal remains.

The ‘Material and methods’ section has now been re-written and presented in a more ‘conventional’ way. The text presented in the section has been moved to Results.

Line 200: The first edition (Russian) of Semenov’s work was published in 1957.

Authors comments: Corrected to ‘1950s’

Lines 213-214: The authors state that ‘the terminology for the different types of knapping tool used in the literature is […] confused.’ Although I agree with them, I wish they would expand more on the issue, and, most importantly, provide clear definition for the terminology they use in their manuscript, as well as how this compares to others, e.g., Mallye et al. 2012; Mozota 2012, 2018, etc.

Authors comments: This sentence has now been removed and a clearer terminology has been added to the methodology section.

Table 2: Please provide the references where the 14C ages were first published.

Authors comments: We have added references to the primary references in the caption and table heading. ‘Other samples’ has been replaced with more specific information on sampling for aDNA.

Line 235: ‘A sample of these features […]’. Please provide an explanation as to how and why this sample of features was selected.

Authors comments: The sentence has been modified to explain that ‘details of these features’ were recorded using the SEM.

Lines 240-241: Please provide a brief summary of the rationale for combining multiple imaging techniques. What type of information do they each provide?

Authors comments: The sentence has been modified to accommodate this suggestion.

Radiocarbon dates: It is not clear to me why the authors didn’t choose to present this information in the “Archaeological context” section. It is only as of line 257 that I realized the 14C ages had been previously published. Perhaps it would be best to reconsider when this paragraph appears in the text.

Authors comments: We have changed the heading to ‘Radiocarbon dating of the knapping tools’. This is part of the results as this is the first time radiocarbon dates have been linked to knapping tools at Gough’s Cave. This is an important result as the dates provide a direct link between these tools to the Magdalenian occupation. This section has now been added under the heading ‘Results’.

Abbreviations: It is not clear why this paragraph doesn’t appear as a Note for Table 2.

Authors comments: This was added according to PLOS ONE's requirements for palaeontology and archaeology research.

Line 324-327: Perhaps it would be good to also mention more recent experimental research on pressure flaking such as Nami and Scheinsohn 1997; Armand and Delagnes 1998; d’Errico et al. 2012; Mozota 2015; Doyon et al. 2019.

Authors comments: We would like to thank the reviewer for suggesting these references which we had overlooked. These have now been read and added to the manuscript.

Lines 333-336: It would be interesting to explain why the authors favor this interpretation as oppose to Jéquier’s according to which these striations may have resulted from preparing the margins of the lithic tools before retouching it (Jéquier et al. 2018)

Authors comments: This has now been added for both the teeth retouchers.

Lines 347-355: This paragraph reads more like a summary of the interpretations. It seems oddly placed as it comes before presenting the results themselves.

Authors comments: Most of this text has now been deleted.

Lines 378-380: Some references supporting this claim would be welcomed.

Authors comments: The appropriate reference is:

Fernández-Jalvo, Y., and Andrews, P., 2016. Atlas of Taphonomic Identifications 1001+ Images of Fossil and Recent Mammal Bone Modification. Vertebrate Paleobiology and Paleoanthropology Series. Springer: Dordrecht, Heidelberg, New York, London.

Line 427: It would be interesting to have a clear image showing an impact that has been sectioned by a fracture and clarify if the fracture is fresh or weathered.

Authors comments: Figure 6 has been modified and a clear photo of the impact has been added (figure 6 C),

Lines 493-495: Please provide references to these experimental studies.

Authors comments: A reference has been provided.

Lines 502-503: Please provide references describing the breakage pattern resulting from each activity and explain how both could be confounded.

Authors comments: The appropriate reference is:

Fernández-Jalvo, Y., and Andrews, P., 2016. Atlas of Taphonomic Identifications 1001+ Images of Fossil and Recent Mammal Bone Modification. Vertebrate Paleobiology and Paleoanthropology Series. Springer: Dordrecht, Heidelberg, New York, London.
Examples of broken metapodials from Gough’s Cave are described in this reference, together with the patterns of breakage inflicted by carnivores of different sizes.

Lines 548 & 558: The interpretation is counter-intuitive. If most knapping damages are concentrated close to the distal end, one would assume the phalanx was held at the proximal end. Furthermore, from the images, despite their low resolution, it would appear the contact with the flint starts from the proximal end toward the distal end and not the other way around. Please clarify.

Authors comments: We have corrected this mistake (line 558) to show that the phalanx was held at the proximal end, as shown in the reconstruction Fig 14B.

Line 576: ‘examind’ should be ‘examined’

Authors comments: Corrected

Line 612: six inches is the equivalent of ~15 cm, not five.

Authors comments: Corrected in text and Fig. 13 caption

Line 693: ‘flacs’ should be ‘flancs’

Authors comments: Corrected

Lines 734-735: Armand and Delagnes (1998) as well as Doyon et al. (2019) make a distinction between active and passive pressure flaking. In their paper, Doyon et al. seem to suggest it would generate different traces which seems to be supported by Mozota (2015) and d’Errico et al. (2012). The use of ‘passive retouching’ could probably be nuanced a little bit here.

T Authors comments: he text has been modified and the references added.

Line 738: Wood can also be used to retouch stone tools. Perhaps citing Martellotta et al. 2021 would be a good idea here.

Authors comments: The Martellotta et al. 2021 reference has been added to line 724

Lines 747-748: Some references of knapping experiments would be appreciated here.

Authors comments: References have been added

Lines 788-789: The use of organic knapping tools in an ad hoc AND curated fashion has been documented elsewhere, e.g., Costamagno et al. 2018; Doyon et al. 2018. It might be worth citing these examples as well.

Authors comments: We checked Costamagno et l. 2018 paper, but we couldn’t find explicit references of curated tools. We noticed that Doyon et al., 2018 also mentioned the presence of *ad hoc* and curated tool at the Lingiing site. However, by writing that ‘..A similar spectrum from simple *ad hoc* knapping tools to curated items is known at least as far back as the early Middle Pleistocene Acheulean..’ we believe we cover other more recent sites.

Lines 799-800: This is more a question than a suggestion, but would you have any idea as to why bone marrow would have been overlooked from these skeletal elements?
Authors comments: No, we can’t say.

Line 816: Here too, Doyon et al. 2018 could be cited.

Authors comments: Interesting question, but this is something we would not be appropriate to address in this paper.

Line 847-849: Jéquier et al. 2018 argue these traces could be resulting from the preparation of the margins of the lithic tools before retouching.

Authors comments: We address this point earlier in the text.

Line 850: Roasting a bone would have made it quite fragile and not very useful for knapping. In Spain, for instance, shepherds are often roasting bones to access the marrow. When exposing a complete bone to the flame, it cracks in less than two or three minutes, easing the access to marrow.

Authors comments: The sentence has been modified to accommodate this suggestion.

Lines 953-954: It is not clear what is implied by the term ‘typical’ here. It could mean either ‘type fossil’ or ‘commonly found’. Needles, lissoirs and retouchers and found in many techno-cultures and therefore and not ‘typical’ of the Magdalenian in the sense of ‘type fossil’. It would perhaps be best to find another word here to avoid ambiguity.

Authors comments: This has been resolved by deleting ‘typical of this techno-culture’.

Line 1032: At the end of this section, I was surprised not to see any comparison with the Pincevent site. Pincevent has been extensively excavated, with a clear demonstration of spatial partitioning of activities (Julien and Karlin 2014) and rare occurrence of bone retouchers (Leroi-Gourhan and Brézillon 1972), which collectively seem to fit what is known for Gough’s Cave.

Authors comments: A discussion of the Pincevent site and material has been added in the discussion.

Line 1044-1047: This is also the case for the Aurignacian, and most of the European Upper Palaeolithic.
Authors comments: We have modified the sentence, but note that the range, diversity and richness of organic artefacts is a feature of the Magdalenian – other Upper Palaeolithic industries have similar types of artefacts, but the Magdalenian takes this to a new level.

**Reviewer #2**: The paper submitted is without major issues. It is of real interest. Here are some minor comments on the manuscript.
l64-66: I do not clearly understand if this information is usefull here? I suggest to delete this sentence.

Authors comments: This sentence links the progression of stone working from the Middle to the Upper Palaeolithic – we modified the sentence and removed the following paragraph break to make this clear.

l86-87: Maybe provide some references on studies where scholars precisely identify the technic of percussion and nature of soft hammer. Furthermore, you provide no indication on the Magdalenian lithic industry of Gough’s Cave: is there no references supporting use of soft organic percussion for at least part of the material?

Authors comments: The technique of percussion is detailed later in the text, both in the methodology and discussion.

Reference to Magdalenian lithics is Jacobi reference [13]

l146-147: I do not understand if this assumption is supported only by anthropological data or if other data support it. If so, maybe provide some references to other arguments.

Authors comments: This statement is based on archaeological data outlined in reference 13. We add this reference and a comment on seasonal use of the cave requested by Reviewer 1.

l216-219: It could be useful to provide the numbers of pieces initially selected after naked eye examination and the proportion of confirmed specimen after binocular and microscopical examination. .

Authors comments: The text now includes a breakdown of the number of pieces examined. This is used to calculate the proportion of the different types of bone used as knapping tools.

l374-376: I did not understood if there are reasons to do not clean pieces to remove this sediment (why not using cleaning bath for example?). Maybe explain that.

Authors comments: We replace ‘adhering’ with ‘concreted’ and we explain why it has not been possible to clean the pieces more thoroughly.

l493-496: Please provide references about these experimental studies

Authors comments: A reference has been added.
